# The Potential of a Digital Twin in Surgery

**DOI:** 10.1177/1553350620975896

**Published:** 2020-12-08

**Authors:** Hanad Ahmed, Laurence Devoto

**Affiliations:** 1 Faculty of Medicine, 7423 University of Southampton, UK; 2 Division of Surgery and Interventional Science, 159099 University College London Medical School, UK

## Introduction

Digital twins are virtual replicas of physical entities that go beyond a still image and encompass the dynamic functionality of the real-life object.^1,2^ They are widely used in industries such as construction and aviation. Their advent is said to mark the fourth industrial revolution for the innovation of new products and services.^1^

The concept is increasingly entering the healthcare industry with the aim of creating molecular and phenotypic copies of human beings that can allow for trialling of different therapies to elucidate the most efficacious treatment for the real-life patient.^2^

Although the literature is increasingly discussing the potential for medical specialities such as cardiology and oncology,^3,4^ there are few articles discussing their potential in surgical practice.

## Digital Twins in Surgical Practice

The ethos of a surgical digital twin is the idea that a patient model is created, and surgery can be planned in the multidisciplinary team meeting, practised beforehand in a simulator and referenced during the operation to verify anatomy and avoid inadvertent damage to structures. This real-time model of the patient could also give rise to clinical trials where new instruments, techniques or therapies are first tried on the digital twin, minimising risk to the patient.

Digital twins combined with the increasingly developing virtual reality platforms can also enhance surgical training for residents, by allowing for simulated practice in the context of each patient’s specific anatomical and physiological variation, whilst providing a realistic account of performance with the ability to measure intraoperative metrics.^5^

## Current Limitations

Current limitations are centred around tissue modelling in real time, with deformation and movement that resembles real life. This issue is mainly one of computer powers as the physics can be modelled using a number of open source programmes.

## Conclusion and Future Practices

Digital twins have the potential to revolutionise surgical care, research and training. Despite the potentials, the healthcare industry is still in its infancy in being able to map the human body down to a dynamic real-time digital counterpart. Even if the technical challenges were to be overcome, there would still be ethical considerations surrounding the ability to download a detailed copy of a human being. Nonetheless, the potential to cure disease in both medicine and surgery is likely to evolve considerably in the coming decades with technology forming a key component of that evolution.
